# The effect of participatory women's groups on birth outcomes in Bangladesh: does coverage matter? Study protocol for a randomized controlled trial

**DOI:** 10.1186/1745-6215-12-208

**Published:** 2011-09-26

**Authors:** Tanja AJ Houweling, Kishwar Azad, Layla Younes, Abdul Kuddus, Sanjit Shaha, Bedowra Haq, Tasmin Nahar, James Beard, Edward F Fottrell, Audrey Prost, Anthony Costello

**Affiliations:** 1 UCL Centre for International Health and Development, Institute of Child Health, 30 Guildford Street, London WC1N 1EH, UK; 2 Perinatal Care Project, Diabetic Association of Bangladesh, 122 Kazi Nazrul Islam Avenue, Dhaka 1000, Bangladesh

**Keywords:** cluster randomised trial, neonatal mortality, community participation, Bangladesh, women's groups

## Abstract

**Background:**

Progress on neonatal survival has been slow in most countries. While there is evidence on what works to reduce newborn mortality, there is limited knowledge on how to deliver interventions effectively when health systems are weak. Cluster randomized trials have shown strong reductions in neonatal mortality using community mobilisation with women's groups in rural Nepal and India. A similar trial in Bangladesh showed no impact. A main hypothesis is that this negative finding is due to the much lower coverage of women's groups in the intervention population in Bangladesh compared to India and Nepal. For evidence-based policy making it is important to examine if women's group coverage is a main determinant of their impact. The study aims to test the effect on newborn and maternal health outcomes of a participatory women's group intervention with a high population coverage of women's groups.

**Methods:**

A cluster randomised trial of a participatory women's group intervention will be conducted in 3 districts of rural Bangladesh. As we aim to study a women's group intervention with high population coverage, the same 9 intervention and 9 control unions will be used as in the 2005-2007 trial. These had been randomly allocated using the districts as strata. To increase coverage, 648 new groups were formed in addition to the 162 existing groups that were part of the previous trial. An open cohort of women who are permanent residents in the union in which their delivery or death was identified, is enrolled. Women and their newborns are included after birth, or, if a woman dies during pregnancy, after her death. Excluded are women who are temporary residents in the union in which their birth or death was identified. The primary outcome is neonatal mortality in the last 24 months of the study. A low cost surveillance system will be used to record all birth outcomes and deaths to women of reproductive age in the study population. Data on home care practices and health care use are collected through interviews.

**Trial registration:**

ISRCTN: ISRCTN01805825

## Background

### The public health importance of addressing neonatal mortality

Every year, 4 million babies die within the first 28 days of life [1]. Another 3 million babies are stillborn, among whom 1 million die during birth [2]. In addition, between 343,000 and 500,000 women die during pregnancy, labour or 42 days post-partum [3,4]. Nearly all of these deaths occur in low and middle income countries. The faster reductions in post-neonatal and child mortality relative to neonatal mortality during the last decades [1] have increased the importance of improving newborn survival to achieve Millennium Development Goal (MDG) 4 (to reduce under-five mortality by two-thirds between 1990 and 2015) [5,6]. Currently, 42% of under-5 deaths occur during the first 28 days of life [2], of which 25-45% within the first 24 hours [1].

While neonatal, and in particular early neonatal, mortality has been relatively resistant to change [1], effective interventions are known for both home and health care settings [7,8]. They include skilled antenatal and delivery care as well as safe home care such as clean delivery practices, breastfeeding and prevention of hypothermia [8]. The scant progress in neonatal survival is contributed to by a lack of evidence on how to deliver effective interventions in contexts where health systems are weak. Every year 60 million women deliver without skilled assistance [9], and maternity care is extremely unequally distributed, with a minority of poor women having access to such care in most low and middle income countries [10]. Evidence is needed on how to improve newborn survival in such contexts.

### Newborn health in Bangladesh

While Bangladesh is on track to achieve MDG4 [11], its burden of neonatal mortality is high. It is the 6^th ^country worldwide with the highest number of neonatal deaths [7]. Around 57% of under-5 deaths in Bangladesh occur in the first month of life [12], of which 74-83% die in the first week [13-15]. About 37 out of 1,000 babies that are born alive die within the first 28 days of life, with infections, low birth weight, and birth asphyxia being the main causes of death [14]. While 60% of pregnant Bangladeshi women make at least one antenatal care visit [12], only 18% of births (13% in rural areas) are assisted by a medically trained provider. This is the lowest coverage worldwide, apart from Ethiopia, Afghanistan and Chad [16]. The vast majority of births (89% in rural areas) is delivered at home, mostly with the assistance of a traditional birth attendant [12].

Government policy on maternal, newborn and child health of newly independent Bangladesh initially focussed on the Expanded Programme on Immunisation, which includes immunisation against tetanus. This led to a major reduction in neonatal tetanus during the late 1970s and 1980s [14]. It was followed in 1998 by the adoption of the Integrated Management of Childhood Illness (IMCI) strategy [17], which, in Bangladesh, includes children from birth till 5 years of age. While IMCI is now in place in most sub-districts, problems remain with the provision of services. In 2009, a National Neonatal Health Strategy and Technical Guidelines have been developed under the stewardship of the Ministry of Health and Family Welfare, underscoring the Government of Bangladesh's commitment to achieve MDG4 [18]. They include recommendations on normal newborn care, neonatal sepsis, low birth weight babies, birth asphyxia, and maternal health. Another national programme geared to reduce maternal as well as neonatal mortality in Bangladesh is the Community Skilled Birth Attendants Programme, in which a cadre of community health workers is being trained to assist home deliveries. A pilot in 2003 has been evaluated as successful and the programme is now being slowly scaled-up [19], but coverage remains low. In addition, several bilateral and non-governmental organisations are active in Bangladesh to improve newborn health.

### Community interventions and the need for further research

Substantial reductions in neonatal mortality can be achieved, even in contexts where health care systems are very weak, as has been shown in cluster randomized trials of community mobilisation with women's groups in rural Nepal and rural tribal areas in India [20,21]. These interventions were modelled on a before-and-after study in remote rural Bolivia, which used a participatory learning and action cycle with women's groups to improve home care practices and health care use [22]. The women's groups, under the guidance of a facilitator, went through a cycle of meetings, in which they identified and prioritized maternal and newborn health problems, and subsequently developed, implemented and evaluated strategies to address these problems, with the support of the entire community. The studies in Nepal and India showed a 30% and 45% reduction in neonatal mortality respectively. There are indications that such participatory strategies for behaviour change are more effective than one-to-one health education [23]. This is consistent with Bandura's social learning theory, which stresses the importance of social interaction, and hence involving the wider community, for behaviour change [24].

There are still questions about the factors that influence the impact of women's group interventions on newborn mortality. The intensity and coverage of community mobilisation might be two such factors. The community mobilisation arm of the Prohjanmo trial in Bangladesh, which was far less intensive than the women's group interventions in Nepal and India, showed no impact on neonatal mortality [25]. A women's group trial running between 2005 and 2007 in rural Bangladesh, of similar high intensity as in Nepal and India also showed no impact on neonatal mortality, or on most home care practices and health behaviours [13]. A main hypothesis is that this was due to the much lower coverage of women's groups in the Bangladesh 2005-2007 trial (1 women's group per 1414 population) compared to Nepal (1:756) and India (1:468). For evidence-based policy making, it is important to better understand these factors. The potential of women's groups to reduce newborn mortality is high and the costs are low [21,26]. Their scalability in the Bangladesh context, with its numerous women's groups involved in a variety of activities such as micro-credit, is high.

This study will examine whether coverage is an important factor influencing the effect of women's groups on neonatal mortality. It will test the effect on neonatal mortality of an intensive women's group intervention with high population coverage.

## Methods/Design

### Objectives and research questions

#### Goal

To improve newborn and maternal health and survival in Bangladesh.

#### Objective

To test the effect on newborn and maternal health outcomes of a participatory women's group intervention with a high coverage of women's groups in the population.

#### Primary research question

What is the effect of a participatory women's group intervention with a high coverage of women's groups on the neonatal mortality rate?

#### Ancillary questions

What is the effect of a participatory women's group intervention with a high coverage of women's groups on:

1. early and late neonatal mortality, stillbirth rate, pregnancy-related mortality ratio, maternal mortality ratio;

2. maternal and newborn home care practices;

3. maternal and newborn health service utilisation.

### Trial design overview

The intervention will be evaluated using a cluster randomized trial design. The rationale is that the intervention is applied to the entire community, involving both regular women's group meetings as well as direct involvement of the wider community. Unions, the lowest administrative level in Bangladesh, are randomized into intervention or control. Because of the nature of the intervention, allocation is not masked. Eighteen unions, 9 intervention and 9 control, with a population of around 28,400 each, are included. The entire study population is around 512,000 people. We have set up a prospective surveillance system in which all live births, stillbirths, neonatal deaths and deaths to women of reproductive age in the study population are recorded and combined with data on home care practices and health care use collected through an interview 6 weeks post-partum [27].

### Setting

**Figure 1:**
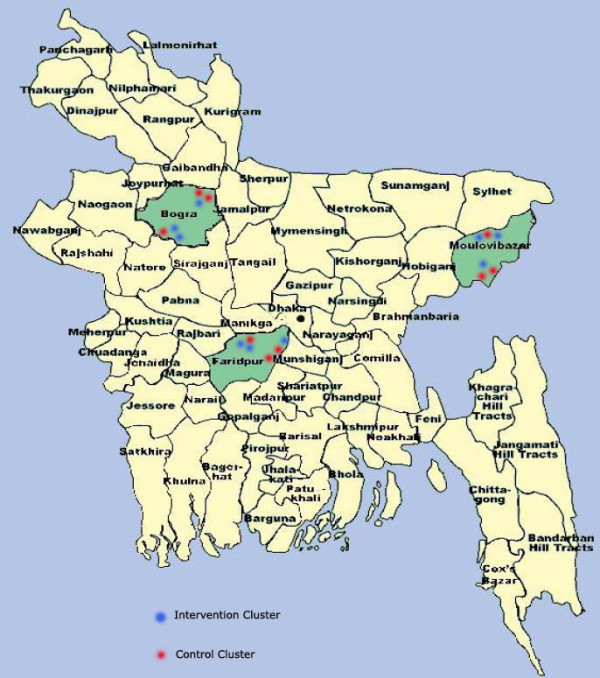
**Study areas**.

The study areas are located in three rural districts of Bangladesh, representing the country's different geographical features [Figure 1]. Moulvibazar district in the northeast is hilly, making travel difficult, and contains socio-economically more deprived tea garden estates with tea garden workers. Faridpur district, to the south of Dhaka, is characterized by large rivers, regularly causing floods and making some unions inaccessible. The study areas in the northern district of Bogra are quite scattered, and recruitment and retention of project staff is comparatively difficult.

Data from our surveillance system show that over 80% of the population in the study areas is Muslim, with nearly all the remainder being Hindu. About half of the women giving birth in the study areas have received no formal education or only primary education. The vast majority of women deliver at home, without professional assistance. In 2007, neonatal mortality was around 35/1,000 in both intervention and control unions. In Bangladesh, primary care is provided at Union Health and Family Welfare Centres and at Community Clinics. In- and out-patient services, including emergency obstetric care, are provided at sub-district (*upazilla*) health complexes and hospitals, and tertiary care is provided at district hospitals and medical college hospitals. In practice, both availability and quality of health care, including maternity care, are important problems in our study areas, due to a lack of facilities and trained health care providers, short supplies of medicines and low responsiveness of services.

The trial is implemented by the Perinatal Care Project (PCP) with technical assistance from the University College London (UCL) Centre for International Health and Development. PCP was established in 2002 as a collaboration between the Diabetic Association of Bangladesh (BADAS), the UCL Centre for International Health and Development (UK), and Women and Children First (UK). In the same study areas, PCP also implemented the 2005-2007 women's group trial, of which the current study is a follow-up.

### Study population

In the intervention areas, the target group for the women's group meetings are permanent residents of reproductive age, in particular pregnant and newly married women. However, other women, such as older in-laws and adolescent girls are also welcome. Community health workers sometimes also participate. Men are allowed, but rarely do attend. The wider community, including both men and women, is involved through, among others, community meetings.

The study population is an open cohort of women, living in the study area, who are permanent residents of the union in which their delivery or death was identified. Women and their newborn infants are included after birth, or, if a woman dies during pregnancy, after her death. A woman is considered a permanent resident of a union if the house she normally lives in is in that union. Excluded are women who are registered as temporary residents in the union in which their delivery or death was identified. These are predominantly women who temporarily move to their parents' house in a different union to give birth, after which they return to their husband's house. On the basis of data from our ongoing surveillance system, we expect 15% of births to occur to women who temporarily move into the PCP study area to give birth, and 6% of births to occur to women who temporarily move to a different union within the PCP study area. Given that 75% of newborn deaths occur during the first week of life, and given the nature of the intervention, in which women's group membership is open to permanent residents and in which entire communities are mobilised to take action during pregnancy, delivery and the newborn period, we expect to see mainly an effect among women who are permanent residents in the union in which they give birth.

### Intervention

#### Community mobilisation through women's groups

The intervention to be evaluated is a participatory learning and action cycle with women's groups. Facilitators, who are local women of reproductive age with at least a high school degree, recruited, trained and paid by PCP, convene monthly women's group meetings. As we aim to study the effect of a women's group intervention with high population coverage, 648 new groups were formed by newly recruited facilitators and started to meet from January 2009 onwards, in addition to the 162 women's groups that were already set up in the intervention areas as part of the 2005-2007 trial [13]. These 'old' groups have continued to meet on a monthly basis from late 2004 onwards [13]. The 648 new groups will go through a cycle of monthly meetings on maternal and newborn health (Cycle 1), while from April 2010 the 162 'old' groups will proceed to a cycle of meetings on under-5 and women's health (Cycle 2) and periodically review maternal and newborn health issues. Membership of the 'old' groups has been relatively stable. The combined 810 women's groups constitute a coverage of 1 group per 300 population, compared to 1 per 1414 in the 2005-2007 trial.

**Figure 2:**
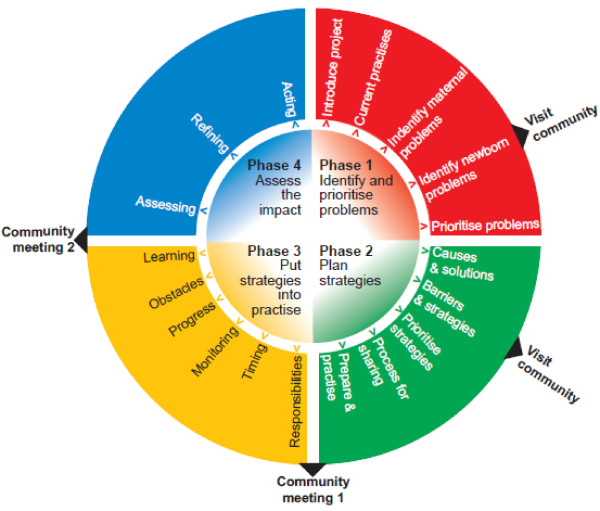
**Community action cycle**.

Each facilitator is responsible for 18 groups. She guides the groups through a four-phased community action cycle [22], in which she activates and supports the groups to identify and prioritize maternal and newborn (cycle 1), or child and women's (cycle 2), health problems (phase 1), plan strategies to address these problems (phase 2), and implement (phase 3) and evaluate (phase 4) these strategies (Figure 2). At the end of phase 2 and at the start of phase 4 community meetings will be held to engage the wider community in the development and implementation of the strategies. The facilitator uses picture cards and a flip chart as means to stimulate discussion. Facilitators received training in participatory communication methods and in basic maternal and newborn (Cycle 1) and child and women's health (Cycle 2) issues. Except for the paid facilitator and tools, no resources are provided to the groups. The groups meet on a monthly basis throughout the intervention period. We expect occasional interruptions of the meeting schedule, for example during the monsoon and Ramadan. Within the 21/2 year period, we expect all groups to have held at least 20 meetings.

The 2005-2007 intervention has been adapted to increase participation of pregnant women in the women's groups. Pregnant women in the community are now identified by facilitators, traditional birth attendants (TBAs), community health workers and women's group members, and are encouraged to attend the groups. Facilitators visit pregnant women and their families to explain the benefits of attending the women's and they keep a record of the number of pregnant women attending the groups.

#### Health systems strengthening

Both intervention and control clusters will receive health system strengthening activities. The reason to include a health systems strengthening component in our study is twofold. First, there is the ethical imperative to ensure that also the control areas benefit from the study. Second, we expect some degree of a functioning local health system to be necessary for the success of a women's group intervention [20]. The health systems strengthening strategy contains four components: sensitisation of community health committees (set up by the Ministry of Health and Family Welfare) to maternal and newborn health issues; basic training of traditional birth attendants in essential newborn care; financial and organisational support of training of doctors; and provision of weighing scales and sphygmomanometers to the 44 community clinics operating in the study area.

### Randomisation

**Figure 3:**
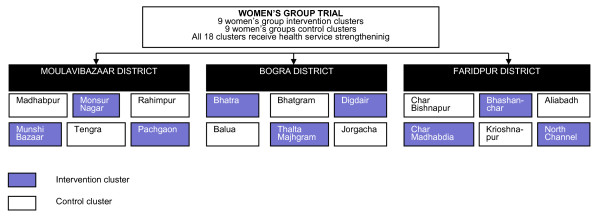
**Randomisation**.

The same intervention and control unions were used as for the 2005-2007 trial. The randomisation for that trial was as follows: the three study districts, Bogra, Faridpur and Moulvibazar, were purposefully sampled on the basis of having active Diabetic Association of Bangladesh (BADAS) offices (Figure 3). In each of these districts, 2 upazillas (sub-districts), and within each upazilla 3 unions were purposefully sampled on the basis of recommendations from BADAS representatives, using perceived limited access to perinatal health care and a feasible travelling distance by motorbike from BADAS district headquarters as the main criteria. The 18 unions were randomly allocated to either intervention or control (9 intervention and 9 control), with each district constituting one stratum, in the presence of four project staff (including the project director and project manager) and two people external to the study team (Dr. Nazmun Nahar, Department of Paediatrics, Dhaka Medical College, Dhaka, and Dr. Azad Khan, BADAS, Dhaka). For each district, cluster names were written on pieces of paper, which were folded and placed in a bottle. The project manager then drew the papers from the bottle. The first three cluster names drawn were allocated to the intervention group and the remaining three to control. The allocation sequence had been decided upon by the project team before drawing the papers [13].

### Reducing contamination

We expect little contamination from the intervention to the control areas. The intervention and control clusters are mostly geographically far apart, though in the few adjacent intervention and control unions, a small number of women from the control areas do participate in the women's group meetings. Mobility, in particular of women of reproductive age, is socially restricted, and opportunities for social mixing between unions are limited. However, a substantial proportion of women temporarily move to their parents' house for delivery. From our previous trial we know these are mostly women arriving from outside the study area. The temporary movement within the study area is expected to be mostly within the same union, rather than between unions. Our study population excludes women that are temporary residents in the union in which their delivery or death was identified.

### Sample size

Sample size was determined following equations from Hayes and Bennett and Hayes and Moulton [28,29], using Microsoft Excel 2003. The trial, with two treatment arms, includes 9 intervention and 9 control areas with an average population of 28,400. The clusters are unmatched. We used 2005-2008 data from our ongoing surveillance system to come to conservative estimates of the number of live births among permanent residents per cluster per year (445), the baseline neonatal mortality rate (34/1,000), and the between-cluster coefficient of variation[28] for neonatal mortality (*k *= 0.099), assuming an equal *k *in intervention and control areas. We assume a 6-month time lag in effect on the basis of findings of similar trials [21]. The study will have between 86% and 90% power to detect a 30% difference in neonatal mortality between the intervention and control arm during the last 24 months of the study. Statistical power remains the same after the exclusion of tea garden residents.

### Impact evaluation

The primary outcome of our study is the neonatal mortality rate (number of deaths in the first 28 complete days after birth per 1,000 live births) in the last 24 months of the study. Secondary outcomes are early and late neonatal mortality rate, stillbirth rate, perinatal mortality rate, pregnancy-related mortality ratio, maternal mortality ratio, and health care use and home care practices during pregnancy, delivery and the neonatal period.

#### Data collection and management

**Figure 4:**
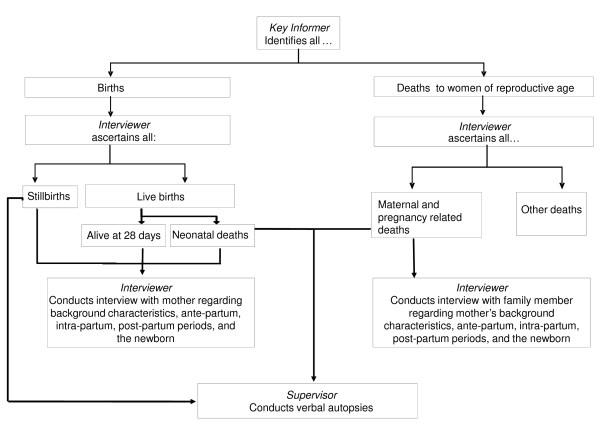
**Surveillance system**.

A low-cost prospective surveillance system will be used to record all deaths during pregnancy up to 6 weeks post-partum and all births and their outcomes (live birth, stillbirth, neonatal death). This system has been in place in the study areas since 2005, and will be maintained throughout the study period (Figure 4). Data are collected using the following steps. 500 key identifiers, all TBAs, are responsible for identifying all women that have given birth and all deaths to women of reproductive age. Each key identifier covers a geographical area of around 200 households. TBAs conduct the majority of deliveries in our study area, and are in a good position to identify births, irrespective of whether they attended them. They are mostly middle-aged women, who are less restricted in movement than newly married women. A full-time salaried monitor (37 monitors work in the study area) meets with the key identifier twice per month to collect the list of identifications. The monitor visits the household to verify the births and deaths, and, for deaths to women of reproductive age, asks if the death occurred during pregnancy up to 6 weeks post partum. The monitor pays the key identifier Tk100 (€1.08, December 2010) for each accurate identification and makes an appointment for the interview. The monitor conducts the interview, which covers background characteristics, and the antenatal, delivery and postpartum periods. In the event of a live birth, stillbirth, or neonatal death, the interview is done with the mother at around 6 weeks post-partum. In the event of a stillbirth or neonatal death, a verbal autopsy is also conducted with the mother. In the case of a death of a woman during pregnancy or up to 6 weeks post-partum, the interview and a maternal verbal autopsy are conducted with a close relative or friend. All eligible women identified are asked if they can identify any other recent deliveries, deaths to women of reproductive age or newborn deaths. Around 20% of questions of 10-20% of all interviews are cross-checked through a re-interview by the monitoring coordinator. Hard copies of the lists of identifications and of the interviews are sent on a monthly basis to the surveillance manager at the PCP head office in Dhaka. The identifications are entered into an SPSS database dedicated to following-up the birth outcomes. The interview data are entered into a Microsoft Access database. The two datasets can be linked using unique identification numbers. All hard copy questionnaires are archived for future reference. Similar surveillance systems are being used in Nepal, India and Malawi [20,27,30,31].

#### Dealing with loss-to-follow-up

For up to 1 year after the birth identification, the monitoring team will seek to obtain information on birth outcomes, pregnancy-related mortality, maternal mortality and health related behaviours, including for mothers who have temporarily or permanently migrated after birth. After one year, they will be considered lost to follow-up. Interviews are arranged at a time that is convenient for the respondent, and monitors are instructed to only conduct an interview if the respondent feels at ease. On the basis of our previous trial, we expect non-response to be very low.

#### Interim analyses and stopping rules

A meeting of an independent Data Safety Monitoring Board (DSMB) will be convened according to the DAMOCLES charter [32], after 11/2 years of accumulated surveillance data. The DSMB will be tasked to provide an independent, objective review of the study implementation and accumulated interim data, and to advise on any extension or modification of the trial. In particular, it will review (i) process indicators including women's group coverage and adherence to the implementation plan, (ii) adequacy of the sample size, (iii) comparability of treatment arms, (iv) data quality, and (v) the proposed analysis plan. The intervention allocation will be masked to the board. There are no stopping rules as we do not expect the intervention to have adverse effects at either cluster or participant level.

#### Analysis plan

The trial will test the effect of a community mobilisation intervention with a high coverage of women's groups. Of the 810 women's groups, 162 are existing groups, which have been meeting from late 2004 onwards, and 648 are new groups, which held their first meeting in January 2009. The 648 new groups focus on pregnancy, delivery and the newborn period (Cycle 1). The 162 old groups focussed on the same until April 2010, after which they started to discuss under-5 and women's health (Cycle 2). The study does not intend to separate out the effects of the old and the new groups, nor of Cycle 1 and Cycle 2. Therefore, any difference in primary and secondary outcomes between intervention and control areas can be due to: (i) increase in coverage of Cycle 1 from 1/1414 to 1/376 (i.e. from 162 to 648 groups), (ii) a 'booster dose' of 162 women's groups with a coverage of 1/1414, (iii) an increase in coverage of women's groups in general (Cycle 1 and 2) from 1/1414 to 1/300. Given that the previous trial had no impact on primary and secondary outcomes and that the focus of Cycle 2 is on the post-neonatal period, we expect any impact on primary and secondary outcomes to be mainly due to increased coverage of Cycle 1.

Analysis will be by intention to treat at the individual and cluster level. Participants are assigned to the cluster in which their birth is registered. The intention-to-treat population only includes women that are permanent residents in the union in which their delivery or death is registered. Participants with missing data on the primary outcome will be excluded from the analyses. The control clusters include three tea garden areas with substantially worse health and socio-economic outcomes compared to the rest of the study area, compromising the comparability of the treatment arms. Analyses will therefore be carried out with and without the tea garden residents. Furthermore, all analyses will be carried out without and with adjustment for potential confounders. Estimates of the intervention effect will be presented with 95% confidence intervals. All analyses will take the clustered design of the study into account. We intend to use STATA-10 to perform the analyses.

As we expect a 6 month time lag for the intervention to take effect, we will use as the trial's primary endpoint the neonatal mortality rate in the last 24 months of the trial.

### Process evaluation

Detailed quantitative and qualitative process evaluation information will be collected throughout the intervention period, to help explain any differences in effect between the old (2005-2007) and the current trial, and formulate alternative hypotheses for the lack of impact of the old trial in comparison with similar trials in India and Nepal. Information will be collected on (1) intervention process, including fidelity to the original intervention protocol, (2) contextual factors that may influence intervention impact, (3) exposure to and participation in the women's group intervention, in particular among pregnant women, and (4) receipt of the intervention by the target population.

### Timetable

The intervention period is between 1 January 2009 and 30 June 2011 (30 months).

### Ethical issues

#### Approvals

The trial has been approved by the University College London Research Ethics Committee (ID Number: 1488/001) and by the Ethical Review Committee of the Diabetic Association of Bangladesh. The trial has been registered with ISRCTN01805825.

#### Community consultation

Community leaders were identified through a resource mapping exercise, and approached to obtain permission to form 648 new women's groups in the existing PCP study areas. Orientation meetings were held to explain the aims of the women's groups and to identify potential women's group members.

#### Consent

Participation in women's groups and community meetings is on a voluntary basis. The participants can choose to leave at any time. Prior to all interviews, the purpose of the data collection is explained and verbal consent from the interviewee obtained. Interviewees are told that they are free to decline the interview, and can refuse to answer any question. Access to the identifiable individual-level data is limited to surveillance and data entry staff and analysts from the study team.

#### Treatment of illness in study areas

Field workers that encounter a sick child or mother are advised to refer them to an appropriate health care facility.

#### Benefits to control areas

We have equipoise on the intervention under test: the effect of an intensive participatory women's group intervention with a high coverage of women's groups is unknown for the Bangladesh context. More generally, it remains unknown if coverage is an important factor determining the magnitude of effect of women's groups on neonatal mortality. Therefore it is important to test the effect of this intervention using a randomized trial design. It is ethically important that the control areas also benefit from the study. They will do so in two ways. First, they will benefit directly, through the health systems strengthening activities that will be undertaken in both study arms. Secondly, they will benefit indirectly, through our advocacy activities at the local, national and international level, and through our surveillance activities, that both are an integral part of our programme. The advocacy activities seek to put maternal and newborn health higher on the political agenda, using health data collected from the study areas.

#### Scalability

The potential for scaling-up a low cost community mobilisation intervention in Bangladesh is high. Scale-up would be facilitated by the fact that PCP is a part of BADAS, the largest health care provider in Bangladesh after the government. BADAS has strong links with the Ministry of Health and Family Welfare and the Ministry of Social Welfare, which can influence the policy making process. Furthermore, the many women's groups and community groups in Bangladesh set up by other organisations, provide a social context which may allow for add-on of a maternal and newborn health component.

#### Role of funder

The funder has no role in the design of the study, the data collection, analysis, interpretation or write-up of the findings.

## List of abbreviations used

BADAS: Diabetic Association of Bangladesh; DSMB: Data Safety Monitoring Board; IMCI: Integrated Management of Childhood Illness; MDG: Millennium Development Goal; PCP: Perinatal Care Project; TBA: Traditional Birth Attendant; UCL: University College London; UK: United Kingdom

## Competing interests

The authors declare that they have no competing interests.

## Authors' contributions

KA is project director, contributed to the design of the study, leads the implementation of the trial, and will participate in the interpretation of data. TH is scientific coordinator of the trial and of the Big Lottery Fund International Strategic Grant. She led the design of the study, wrote the first draft of the study protocol, and will participate in the analysis and interpretation of data. AK is project manager of the trial, leads the health systems strengthening activities, and will participate in the interpretation of data. LY is research assistant to the study, contributed to the design of the intervention, and will participate in the analysis and interpretation of data. SS coordinates the data collection and will participate in the analysis and interpretation of data. JB provides technical support to the data collection and data management, and will participate in the analysis of the data. TN contributed to the design of the intervention, is responsible for the implementation of the women's groups intervention, and will participate in the interpretation of the data. BH is responsible for the process evaluation and will participate in the analysis and interpretation of the data. AP, AC and EF contributed to the design of the study and will participate in the analysis and interpretation of data. AC and KA obtained the funding for the study. All authors have contributed to and agreed to the final version of the manuscript.
